# Healthcare experiences and expectations of women survivors of domestic violence in Asia: a systemized review of the literature

**DOI:** 10.1080/16549716.2026.2721051

**Published:** 2026-08-27

**Authors:** Md. Khobair Hossain, Jo Brown, Helen Ansell

**Affiliations:** Institute of Health and Allied Professionals, Nottingham Trent University, Nottingham, UK

**Keywords:** Intimate partner violence, trauma informed care, help-seeking behavior, health system responsiveness, culturally sensitive care, domestic violence

## Abstract

Domestic violence significantly shapes women’s healthcare experiences and expectations in Asian settings; however, evidence remains dispersed across diverse study designs and countries. This review synthesizes empirical findings on how survivors navigate healthcare systems and what they expect from providers. A systematized review was conducted across PubMed, Scopus, Web of Science, CINAHL, and Library OneSearch databases. Eligible studies were published between 2000 and May 2024, conducted in Asian countries, and reported on women’s healthcare experiences and/or expectations in the context of domestic or intimate partner violence. Qualitative, quantitative, and mixed-methods studies were included. Quality appraisal was conducted using CASP and MMAT, and findings were synthesized narratively. The literature search was conducted in June 2024 and included studies published up to May 2024. Fifteen studies met the inclusion criteria and represented diverse Asian settings. Findings indicate that cultural stigma, fear of retaliation, and victim-blaming contribute to non-disclosure and avoidance of healthcare. Survivors reported expectations for empathetic communication, confidentiality, and culturally sensitive care; however, institutional limitations, provider constraints, and inconsistent referral pathways often hindered the fulfilment of these expectations. Quantitative findings further indicate reduced service utilization among survivors and significant associations between domestic violence and adverse mental health outcomes. Across Asian contexts, survivors’ expectations of safe, confidential, and supportive care are frequently unmet due to structural, cultural, and provider-level barriers. Strengthening trauma-informed, culturally sensitive healthcare responses and improving referral pathways may enhance disclosure, continuity of care, and safety for affected women.

## Paper Context

- **Main findings:** Women survivors of domestic violence in Asian settings experience substantial barriers to accessing safe, confidential, and responsive healthcare services.
- **Added knowledge:** This review shows how cultural norms, stigma, and provider practices shape both healthcare experiences and expectations of survivors across diverse Asian contexts.
- **Global health impact for policy and action:** Strengthening culturally responsive, trauma-informed, and integrated healthcare systems is essential to improve access, safety, and quality of care for survivors.

## Background

Domestic violence (DV), particularly intimate partner violence (IPV), is a pervasive global public health issue with profound implications for women’s physical and mental health. The World Health Organization (WHO) estimates that approximately 30% of women worldwide experience physical or sexual violence by an intimate partner during their lifetime [1]. The health consequences of DV are extensive, ranging from acute physical injuries, such as fractures and lacerations, to chronic conditions including gastrointestinal and reproductive health problems, as well as mental health outcomes such as anxiety, post-traumatic stress disorder (PTSD), and suicidal ideation [2,3].

Although DV affects individuals across all regions and demographics, its prevalence and manifestations vary significantly by geography and culture. For example, reports from the European Union estimate IPV prevalence at around 22% [4], whereas studies from South and West Asia indicate substantially higher rates. In Jordan, up to 69% of married women of reproductive age report experiencing physical violence, while in Iraq, the figure is estimated at 52% [5]. These disparities underscore the need for region-specific analyses that account for the unique socio-cultural dynamics influencing both the occurrence of DV and survivors’ interactions with healthcare systems.

This review focuses on the healthcare experiences and expectations of women survivors of domestic violence (DV) in selected Asian contexts, particularly South, East, and West Asia. The rationale for this focus is based on both epidemiological and socio-cultural factors. Women in these regions face entrenched patriarchal norms, honour-based stigma, and collectivist family structures that often prioritize family reputation over individual well-being [6,7]. These conditions create major barriers to help-seeking, including fear of disclosure, victim-blaming by healthcare providers (HCPs), and limited autonomy in healthcare decision-making.

Although DV affects men and individuals of diverse gender identities, this review focuses specifically on women survivors due to their higher exposure to IPV and the gendered nature of healthcare barriers in Asian settings. Women’s help-seeking behavior is often shaped by social expectations of silence and endurance, limited access to female HCPs, and cultural taboos around discussing abuse [2,7]. These challenges are less common in many Western healthcare settings, where systems for responding to DV are generally more developed [8].

Despite the widespread nature of DV and its serious health impacts, seeking healthcare remains difficult for many survivors. Differences in healthcare use are clear; for example, help-seeking rates range from 87% in Jordan to 25.7% in Modena City, Saudi Arabia [9,10]. In South Asia, barriers such as stigma, lack of privacy, and provider insensitivity further limit access to care [11]. In addition to physical injuries, DV causes a significant mental health burden, highlighting the need for healthcare approaches that integrate both physical and psychological care [12,13].

Existing healthcare services for survivors of domestic violence (DV) are often criticized for not fully addressing their needs and expectations. Traditional healthcare models, typically based on biomedical approaches, may not adequately respond to the complex and culturally sensitive nature of DV [14]. These models often overlook trauma-informed care and fail to consider the cultural importance of family honour, which can strongly discourage disclosure and engagement with healthcare services [15].

For example, in rural Northeast India, women reported experiencing obstetric violence during hospital births, showing how institutional practices can continue to cause harm even in settings meant to ensure safety [16]. Similarly, in Malaysia and Palestine, survivors described dismissive attitudes and lack of privacy during consultations, which discouraged future help-seeking [17–20].

Despite these challenges, there is still limited consolidated evidence on how women survivors experience and navigate healthcare systems in Asian contexts.

The primary aim of this study is to synthesize existing literature on the healthcare experiences and expectations of women survivors of domestic violence in South, East, and West Asia. The review addresses the following question: What are the healthcare experiences and expectations of women experiencing domestic violence in Asian countries, as reported in qualitative and observational studies?

By focusing on survivors’ perspectives, this review provides a consolidated evidence base to inform healthcare policy, service development, and practice. It aims to support the development of culturally sensitive, trauma-informed, and survivor-centred healthcare approaches that address systemic barriers, improve provider training, and strengthen community engagement. Ultimately, the review seeks to inform the development of more equitable and effective care models for this population.

## Methods

### Study design

This study employed a systematized literature review approach, combining elements of systematic and narrative reviews to allow for a structured, yet flexible, synthesis. The review followed a predefined protocol for database searching, inclusion criteria, and thematic analysis, although it was not formally registered. This approach was chosen to accommodate the diversity of study designs and the culturally embedded nature of domestic violence in Asian contexts [21].

This review was conducted and reported in accordance with the PRISMA 2020 guidelines, and a PRISMA checklist is provided as a supplementary file. The study followed predefined search strategies, inclusion and exclusion criteria, and a structured thematic synthesis. However, the review was not prospectively registered in a database such as PROSPERO. This reflects the systematized review design adopted within the scope of a graduate-level dissertation and should be considered when interpreting the findings.

### Search strategy

A three-phase search strategy was used to identify relevant literature published between January 2000 and May 2024. In the first phase, broad and specific keywords were developed based on research questions and existing literature on domestic violence and healthcare access. These included terms such as ‘domestic violence,’ ‘intimate partner violence,’ ‘women,’ ‘healthcare experiences,’ ‘expectations,’ ‘Asia,’ and ‘cultural barriers.’

In the second phase, searches were conducted across five major bibliographic databases: PubMed, Scopus, Web of Science, CINAHL, and Library OneSearch (Nottingham Trent University Library). These databases were selected to ensure comprehensive coverage of health, nursing, and social science literature. Google Scholar was also used to identify grey literature and studies from regional or interdisciplinary sources that may not be indexed in conventional databases.

In the third phase, the reference lists of all included articles were manually reviewed to identify additional studies that met the inclusion criteria but were not captured in the initial database searches.

### Inclusion and exclusion criteria

Studies were included if they were published in peer-reviewed journals between 2000 and May 2024, written in English, and conducted in Asian settings, specifically South and West Asia. Eligible studies used qualitative, quantitative, or mixed methods designs and examined the healthcare experiences and/or expectations of adult women who had experienced domestic violence.

Studies were excluded if they lacked sufficient data richness. This was defined as the absence of detailed findings, limited use of direct participant quotations, or superficial analysis that limited meaningful thematic synthesis [22,23]. Studies conducted outside the defined geographical scope or those not focused on healthcare interactions were also excluded.

### Study selection process

The initial search identified 334 articles. After removing 37 duplicates, 297 titles and abstracts were screened, of which 189 were excluded as not relevant. Of the remaining 108 full-text articles assessed for eligibility, 93 were excluded for the following reasons: methodological irrelevance (*n* = 8), insufficient data richness (*n* = 69), and location outside the defined geographical scope (*n* = 16).

**Figure 1.**
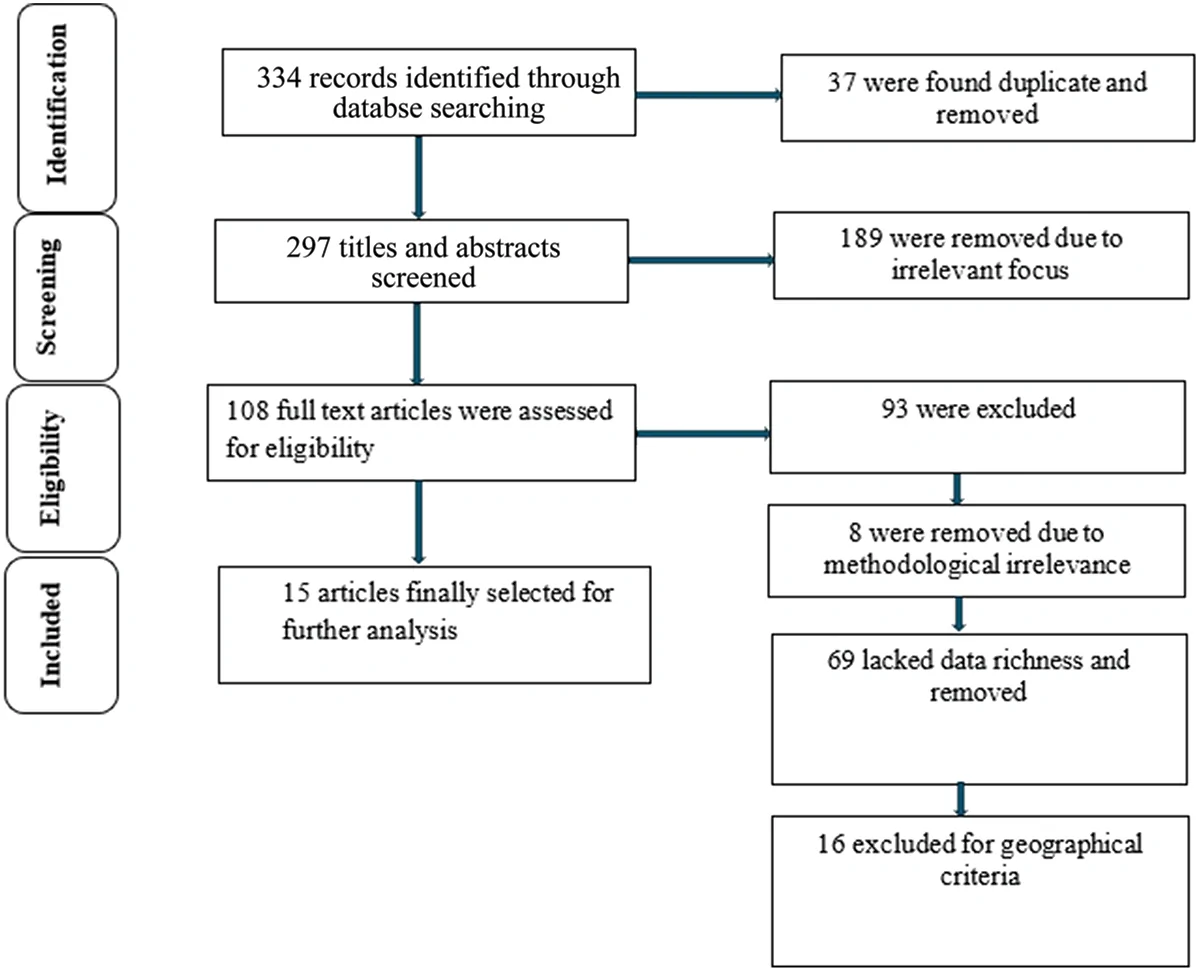
Study selection process for the systematized review. This figure presents the study selection process used in this systematized review. A total of 334 records were identified through database searching. After removing 37 duplicates, 297 titles and abstracts were screened, of which 189 records were excluded as not relevant. A total of 108 full-text articles were assessed for eligibility, and 93 were excluded due to methodological irrelevance (n = 8), insufficient data richness (n = 69), or location outside the defined geographical scope (n = 16). Fifteen studies met the inclusion criteria and were included in the final synthesis.

This process resulted in a final selection of 15 studies for inclusion in the review (Table 1; Figure 1).

**Table 1.** Characteristics and quality appraisal of included studies.

Study (first author, year)	Country / setting	Design	Sample size	Methods (data collection)	Appraisal tool	Quality category	Key limitations / risk of bias	Journal
Chuah et al., 2022	Malaysia (providers)	Qualitative (descriptive)	n = 20	Semi-structured interviews	CASP	Moderate concerns	Providers only; survivor perspective absent → potential information bias	Int J Equity Health
Barez et al., 2023	Iran	Qualitative (descriptive)	n = 25	Semi-structured interviews	CASP	Moderate concerns	Privacy procedures not fully detailed; disclosure may be constrained	BMC Women’s Health
Islam et al., 2018	Bangladesh	Mixed methods	n ≈ 20 + unspecified	In-depth & informal interviews; key informants; FGDs; documents	MMAT (2018)	Moderate concerns	FGD participants not fully specified → information bias	Trop Med Health
Sattar et al., 2022	Pakistan (Southern Punjab)	Qualitative (phenomenological)	n = 46	In-depth / semi-structured interviews	CASP	Moderate concerns	Translation process unclear → possible translation bias	BMC Women’s Health
Chahal, 2015	Pakistan (clinical)	Qualitative (focused ethnography)	n = 37	Semi-structured interviews; FGDs; observation	CASP	Moderate concerns	Researcher presence may alter behaviour (observer effect)	BMJ Sex Reprod Health
Silva et al., 2022	Sri Lanka	Qualitative	n = 20	In-depth interviews	CASP	Moderate concerns	Retrospective narratives → recall bias	BMC Public Health
Chattopadhyay et al., 2018	India (Northeast)	Qualitative	n = not reported	Interviews	CASP	Moderate concerns	Potential observer effect; sample size not reported	Cult Health Sex
van der Putten & NurEJannat, 2022	Bangladesh	Qualitative (case study)	n = 25	Case-based interviews	CASP	Moderate concerns	Purposive selection → selection bias risk	J Health Res
Othman et al., 2014	Iraq (clinical)	Qualitative	n = 10	Interviews	CASP	Moderate concerns	Victims only; one-sided reporting bias	J Interpers Violence
Shaheen et al., 2020	Palestine	Qualitative	n = 20	Interviews	CASP	Moderate concerns	Recall and social desirability bias	BMC Public Health
Dandona et al., 2022	India (multi-state)	Quantitative (time series)	n > 100,000	Secondary data; time series	MMAT (2018)	Moderate concerns	Data quality varies; generalisability issues	BMC Women’s Health
Liu et al., 2023	South Asia	Quantitative (cross-sectional)	n = 4,467	DHS-based survey	MMAT (2018)	Moderate concerns	Measurement bias (social norms)	J Glob Health
An et al., 2024	South Korea	Quantitative (survey)	n = 600	Online survey; logistic regression	MMAT (2018)	Moderate concerns	Self-report; limited population	PLoS One
Kita et al., 2022	Japan	Qualitative (narrative)	n = 23 (11 analyzed)	Narrative interviews	CASP	Moderate concerns	Only partial sample analysed; recall bias	Int J Environ Res Public Health
Thitiyanviroj et al., 2024	Thailand (hospital-based)	Qualitative (FGDs)	n = 32	Focus group discussions	CASP	Moderate concerns	Group dynamics; confidentiality concerns	Nurs Womens Health

Note: Quality appraisal categories were defined as follows: High quality-studies meeting most CASP/MMAT criteria with minimal risk of bias; Moderate concerns-studies with some methodological limitations but overall acceptable rigor; Significant concerns-studies with multiple methodological limitations that may affect confidence in findings. No numerical scoring was applied in accordance with MMAT guidance.

### Quality appraisal

To enhance methodological rigor, the included studies were assessed using established appraisal tools. Qualitative studies were evaluated using the Critical Appraisal Skills Program (CASP) checklist, while quantitative and mixed-methods studies were assessed using the Mixed Methods Appraisal Tool (MMAT) [24,25].

In accordance with MMAT guidance, numerical scoring was not applied. Instead, studies were appraised across relevant methodological domains.

Based on the extent to which these criteria were met, studies were categorized as ‘high quality,’ ‘moderate concerns,’ or ‘significant concerns.’ These classifications were used to support the interpretation of findings rather than to exclude studies from the synthesis. Detailed study-level appraisal outcomes are presented in Table 1.

### Data extraction and analysis

Data were extracted systematically from each included study using a pre-designed extraction form. This form captured key information, including country of origin, study design, participant characteristics, healthcare setting, and findings related to healthcare experiences and expectations.

Data extraction and initial coding were conducted by the primary reviewer. To strengthen methodological rigor, a subset of studies was reviewed and discussed with an academic supervisor experienced in qualitative research and evidence synthesis. This process helped verify coding decisions, resolve ambiguities, and refine the thematic framework. Given the single-author design of this systematized review, the supervisory role was advisory and confirmatory rather than constituting independent dual data extraction.

During data extraction, we also assessed whether multiple included studies were derived from the same dataset or study cohort. When overlaps were identified, articles were retained only if they addressed different research questions, outcomes, or analytical perspectives relevant to this review. Evidence from such studies was synthesized at the dataset level rather than treated as independent observations, to avoid double counting. Where overlap was substantial, findings were consolidated and interpreted as complementary rather than additive contributions.

Thematic analysis was conducted using an inductive and iterative approach informed by descriptive phenomenology [23]. Open coding was applied to relevant data segments, and emerging codes were grouped into broader themes aligned with the research questions. A cultural theoretical lens was applied throughout the analysis to interpret how survivors’ healthcare experiences and expectations were shaped by socio-cultural norms, gender dynamics, and healthcare system structures [26,27].

### Thematic framework

The final themes were organized into two overarching categories: healthcare experiences and healthcare expectations. The ‘experiences’ category captures survivors’ interactions with healthcare providers, including barriers to disclosure, perceptions of care quality, and emotional responses to treatment. The ‘expectations’ category reflects survivors’ anticipated needs and preferences, such as privacy, empathy, professionalism, and safety.

This categorization highlights the gap between the care survivors receive and the care they expect and provides a clearer understanding of how past experiences influence future help-seeking behavior and perceptions of healthcare systems.

## Results

This systematized review synthesizes findings from fifteen peer-reviewed studies conducted across South, West, and East Asia. The results are presented in three sections: study selection, study characteristics, and thematic analysis of healthcare experiences and expectations.

### Study selection

A comprehensive search identified studies examining the healthcare experiences and expectations of women affected by domestic violence in Asian contexts. After removing duplicates, titles and abstracts were screened, followed by full-text assessment based on predefined inclusion criteria. Fifteen studies met the eligibility criteria and were included in the final review. The selection process is summarized in Table 1, which presents the number of records identified, screened, excluded, and included.

### Study characteristics

The included studies varied in design, with most using qualitative approaches such as phenomenological, ethnographic, and descriptive methods, while others employed mixed-methods or quantitative designs. Data collection methods included in-depth interviews, semi-structured interviews, focus group discussions, and surveys.

Sample sizes ranged from 10 participants in qualitative studies to over 100,000 in large-scale quantitative analyses. Studies were conducted across diverse Asian settings, reflecting cultural and health system differences. Common limitations included recall bias, social desirability bias, and sampling bias, which may affect interpretation and generalizability.

Overall, most studies were assessed as high quality or as having moderate methodological concerns, with only a small number classified as having significant concerns.

### Healthcare experiences

This section synthesizes findings related to women’s experiences of interacting with healthcare systems following domestic violence. Both qualitative and quantitative evidence are presented to capture key aspects of care, including disclosure, provider response, adequacy of services, and health outcomes. Qualitative themes drawn from survivors’ narratives are summarized in Table 2., while quantitative findings on healthcare utilization and mental health outcomes, interpreted here as indicators of insufficient care and undiagnosed trauma, are presented in Table 3.

**Table 2.** Healthcare experiences of women victims of domestic violence.

Theme / Code	Definition	Representative evidence	Studies contributing (n)
Fear of disclosure & stigma	Stigma, victim-blaming and fear of retaliation discourage disclosure in clinical encounters	‘I felt like I would be blamed … so that would make me shy, embarrassed to talk about it.’ (Palestine)	n = 9 Chuah et al., 2022 (Int J Equity Health) Islam et al., 2018 (Trop Med Health) Sattar et al., 2022 (BMC Women’s Health) Shaheen et al., 2020 (BMC Public Health) Barez et al., 2023 (BMC Women’s Health) Chattopadhyay et al., 2018 (Cult Health Sex) van der Putten & Nur-E-Jannat, 2022 (J Health Res) Othman et al., 2014 (J Interpers Violence) Kita et al., 2022 (Int J Environ Res Public Health)
Confidentiality & trust gaps	Concerns that disclosures will not be kept private or acted upon appropriately	Survivors expressed skepticism about privacy protections and feared secondary harm (Bangladesh)	n = 10 Shaheen et al., 2020 (BMC Public Health) Chattopadhyay et al., 2018 (Cult Health Sex) Chuah et al., 2022 (Int J Equity Health) Islam et al., 2018 (Trop Med Health) Sattar et al., 2022 (BMC Women’s Health) Barez et al., 2023 (BMC Women’s Health) Chahal, 2015 (BMJ Sex Reprod Health) Silva et al., 2022 (BMC Public Health) Kita et al., 2022 (Int J Environ Res Public Health) Thitiyanviroj et al., 2024 (Nurs Women’s Health)
Rushed or insufficient care	Short consultations and limited follow-up reduce survivor-centred responses	‘She works fast … I didn’t feel like the doctor would listen to me.’ (Palestine)	n = 6 Islam et al., 2018 (Trop Med Health) Sattar et al., 2022 (BMC Women’s Health) Shaheen et al., 2020 (BMC Public Health) Chahal, 2015 (BMJ Sex Reprod Health) Kita et al., 2022 (Int J Environ Res Public Health) Liu et al., 2023 (J Glob Health)
Psychological sequelae under-addressed	Emotional and mental-health needs often overlooked relative to visible injuries	IPV associated with depressive symptoms; PTSD narratives common (Japan)	n = 8 Kita et al., 2022 (Int J Environ Res Public Health) Chahal, 2015 (BMJ Sex Reprod Health) Silva et al., 2022 (BMC Public Health) Thitiyanviroj et al., 2024 (Nurs Women’s Health) Islam et al., 2018 (Trop Med Health) Sattar et al., 2022 (BMC Women’s Health) Shaheen et al., 2020 (BMC Public Health) An et al., 2024 (*PLoS One*)
Empowering encounters	Empathy, validation, and consent-seeking build trust and promote help-seeking	Respectful communication increased willingness to return (Palestine)	n = 4 Shaheen et al., 2020 (BMC Public Health) Chuah et al., 2022 (Int J Equity Health) Islam et al., 2018 (Trop Med Health) Dandona et al., 2022 (BMC Women’s Health)

**Table 3.** Summary of quantitative evidence on healthcare utilization and mental health outcomes among women exposed to intimate partner violence.

Study (Ref.)	Country / Region	Outcome(s) Examined	Effect Estimate(s)	Notes
Liu et al., 2023 [Figure 1 Resub | Word]	South Asia	Antenatal care utilization among women exposed to less severe physical violence	OR = 0.55; 95% CI 0.40–0.76; *p* < 0.001	IPV exposure associated with *lower* ANC use
An et al., 2024 [Figure 1 Resub | Word]	South Korea	Depressive symptoms among IPV-exposed young adults	OR = 1.05; *p* < 0.05	IPV associated with increased depressive symptoms
Liu et al., 2023 (descriptive) [Figure 1 Resub | Word]	South Asia	Prevalence of psychological abuse vs. physical injury	Psychological abuse 24.59%; physical injury 11.67%	Highlights under-detected psychological IPV
Dandona et al., 2022 [Figure 1 Resub | Word]	India (multi-state)	Trend analysis of DV burden (time-series)	No OR reported (ecological trends)	Retained for contextual quantitative value

### Fear and stigma

Survivors across multiple contexts reported experiences of fear and stigma when engaging with healthcare services. Women described concerns about being blamed, judged, or socially exposed if they disclosed violence during healthcare encounters. A participant from Palestine stated, ‘I felt like I would be blamed for it … people might say, “look, she revealed what happens between her and her husband. Why would she say that?” This made me feel shy and embarrassed to talk about it’ [28]. Such internalized stigma, combined with external judgment, created significant barriers to accessing care.

In Thailand, providers reported that screening for intimate partner violence was often limited, as violence was commonly viewed as a private family matter [29]. These perceptions were associated with reduced inquiry into domestic violence during clinical consultations.

### Trust and confidentiality

Trust and confidentiality were frequently reported as key aspects of survivors’ healthcare experiences. Many women expressed uncertainty about whether their disclosures of domestic violence would be kept private or taken seriously by healthcare providers. In Bangladesh, survivors reported concerns that personal information shared during consultations might be disclosed without consent, leading to further harm or social consequences [30]. Participants also described previous encounters in which provider responses were perceived as unsupportive or dismissive.

### Insufficient care and undiagnosed trauma

Survivors across multiple regions described receiving care they perceived as insufficient or incomplete. In South Asia, women reported long waiting times, brief consultations, limited engagement from providers, and a lack of follow-up care [31]. Similar experiences were also reported in West Asian settings. One participant from Ramallah shared, ‘I didn’t feel like the doctor would listen to me … there were many people waiting, and she was working very quickly’ [28,31]. Survivors frequently reported short consultation times and limited interaction with healthcare providers.

Quantitative findings were presented using consistent reporting of effect estimates. Among pregnant women in South Asia, exposure to less severe physical violence was associated with reduced antenatal care utilization (OR = 0.55; 95% CI 0.40–0.76; *p* < 0.001) [13]. In East Asia, intimate partner violence was significantly associated with increased depressive symptoms among young adults (OR = 1.05; *p* < 0.05) [13]. In national-level analyses from South Asia, psychological abuse was more prevalent (24.59%) than physical injury (11.67%), highlighting differences in the visibility and health system detection of IPV-related harm [13]. A summary of all quantitative effect estimates is provided in Table 2 and 4.

**Table 4.** Healthcare expectations of women victims of domestic violence.

Theme / Code	Definition	Representative evidence	Studies contributing (n)
Privacy & safe spaces	Expectation for private, gender-sensitive clinical environments to discuss DV safely	Preference for female providers and private consultation spaces reported across multiple Asian settings	n = 12 Sattar et al., 2022 (BMC Women’s Health) Shaheen et al., 2020 (BMC Public Health) An et al., 2024 (PLoS One) Silva et al., 2022 (BMC Public Health) Chuah et al., 2022 (Int J Equity Health) Dandona et al., 2022 (BMC Women’s Health) Islam et al., 2018 (Trop Med Health) Chattopadhyay et al., 2018 (Cult Health Sex) Barez et al., 2023 (BMC Women’s Health) Chahal, 2015 (BMJ Sex Reprod Health) Kita et al., 2022 (Int J Environ Res Public Health) Liu et al., 2023 (J Glob Health)
Trauma-informed, empathetic care	Expectation for non-judgemental listening, validation, and emotional support	Survivors sought compassionate communication, reassurance, and dignity-affirming interactions	n = 10 Islam et al., 2018 (Trop Med Health) An et al., 2024 (PLoS One) Barez et al., 2023 (BMC Women’s Health) Silva et al., 2022 (BMC Public Health) Chuah et al., 2022 (Int J Equity Health) Dandona et al., 2022 (BMC Women’s Health) Chattopadhyay et al., 2018 (Cult Health Sex) Sattar et al., 2022 (BMC Women’s Health) Shaheen et al., 2020 (BMC Public Health) Chahal, 2015 (BMJ Sex Reprod Health)
Routine identification & referral	Expectation that providers will screen for IPV and offer clear referral pathways	Anticipation of proactive enquiry and structured linkage to appropriate services	n = 7 Kita et al., 2022 (Int J Environ Res Public Health) Liu et al., 2023 (J Glob Health) Chuah et al., 2022 (Int J Equity Health) Shaheen et al., 2020 (BMC Public Health) An et al., 2024 (PLoS One) Islam et al., 2018 (Trop Med Health) Chattopadhyay et al., 2018 (Cult Health Sex)
Holistic, integrated support	Expectation for coordinated physical, psychological, legal, and social support	Desire for referral to counselling, shelters, legal aid, and community resources	n = 8 Shaheen et al., 2020 (BMC Public Health) Dandona et al., 2022 (BMC Women’s Health) An et al., 2024 (PLoS One) Chuah et al., 2022 (Int J Equity Health) Liu et al., 2023 (J Glob Health) Sattar et al., 2022 (BMC Women’s Health) Islam et al., 2018 (Trop Med Health) Barez et al., 2023 (BMC Women’s Health)
Respect for autonomy	Expectation that providers respect women’s decisions and self-determination (e.g. leaving the relationship)	‘It was fine to be me even if I am not perfect.’ (Japan)	n = 6 Islam et al., 2018 (Trop Med Health) Barez et al., 2023 (BMC Women’s Health) An et al., 2024 (PLoS One) Silva et al., 2022 (BMC Public Health) Chattopadhyay et al., 2018 (Cult Health Sex) Shaheen et al., 2020 (BMC Public Health)

In Japan, one study documented the long and non-linear recovery journeys of survivors of intimate partner violence [32]. Among eleven participants, eight had been diagnosed with psychiatric conditions, including post-traumatic stress disorder and mood disorders. One participant described her experience during the ‘Burning Out’ phase as follows: ‘I had stomach pain, palpitations, and felt cold all the time. I thought I might die soon’ [32]. Participants also reported experiencing physical symptoms that were not initially linked to their experiences of violence during healthcare encounters. Many described a separation between physical symptoms and psychological distress.

Several women later reported recognizing connections between their physical symptoms and past trauma through peer support, personal reflection, or self-education, rather than through clinical explanation. One participant stated, ‘I realized my uterine pain and tears were connected to the rape. No doctor ever told me that’ [32].

### Empowerment and validation

Some survivors described positive healthcare experiences. Participants reported respectful communication, shared decision-making, and careful documentation of injuries during consultations. Survivors noted that providers who sought consent before examinations and showed empathy were viewed positively. One participant from Ramallah stated, ‘She asked me, “What’s going on? I feel like you’re not all right.” I told her, ‘I’m having some problems with my husband’ [28].

In Japan, survivors also described aspects of recovery that involved recognizing connections between physical and psychological experiences [32]. These insights were often developed through peer support or personal reflection, rather than through clinical encounters [32].

### Healthcare expectations

Findings related to women’s expectations of healthcare systems and providers are presented separately to distinguish anticipated needs from lived experiences. These expectations include safety, confidentiality, respectful communication, cultural sensitivity, and coordinated care. Themes derived from qualitative studies are summarized in Table 4, highlighting both alignment and gaps between survivors’ expectations and actual healthcare responses across settings.

### Safe accessibility and privacy

Survivors expected healthcare environments to be safe and private. Participants reported a preference for easy access to services, gender-separated counselling, and respectful treatment during consultations. In Palestine, women emphasized the importance of female providers and private spaces for discussing sensitive issues, and reported discomfort during consultations with male providers [28,33].

In East Asia, a quantitative study reported associations between intimate partner violence perpetration and certain health-related factors, including alcohol use (OR = 1.23, *p* < .05) and physical health status (OR = 0.89, *p* < .01) [13]. These findings were presented in relation to IPV exposure and healthcare experiences.

### Trust and confidentiality

Survivors expected healthcare providers to maintain confidentiality during consultations. Participants emphasized the need for reassurance that disclosures would be handled privately and respectfully, and that personal information would not be shared without consent. These expectations were reported across multiple settings [33,34].

### Professionalism and empathy

Survivors highlighted the importance of professional conduct and empathetic engagement during healthcare encounters. They valued adherence to clinical protocols, emotional support, and clear, respectful communication from providers [33,34]. In Thailand, both survivors and healthcare providers called for improved IPV screening practices and better provider training [38]. Similarly, participants in a Korean study expected healthcare encounters to be respectful and responsive to their needs [35].

In Japan, survivors described expectations related to validation and respect during later stages of recovery. Participants expressed a desire for providers to acknowledge their personal decisions, including decisions to leave abusive relationships, and to treat them with dignity [32]. One participant reflected, ‘It was fine to be me even if I am not perfect’ [28]. These expectations were expressed in narratives describing interactions with healthcare providers.

### Safety and support

Survivors expected healthcare settings to provide safety and access to support services. Participants in South and East Asian contexts reported dissatisfaction with responses from family members and community networks and described turning to healthcare services for support and guidance [36–38]. Survivors also expected providers to offer assistance, share information about available services, and facilitate connections to social and legal resources.

## Discussion

This systemized review examines how cultural norms, gender expectations, and social hierarchies shape the healthcare experiences and expectations of women affected by domestic violence (DV) in Asian settings. Across the included studies, survivors’ engagement with health services was influenced by family honor norms, expectations of silence, and community-level moral judgments, creating barriers to disclosure, timely help-seeking, and perceived quality of care [39,40].

A recurring theme was the normalization of DV as a ‘private’ or ‘domestic’ matter, discouraging women from seeking external support. Women in Palestine reported fear of blame and social embarrassment when considering disclosure in clinical settings [41], while similar patterns were observed in Bangladesh and Pakistan, where survivors anticipated stigma and community backlash [42]. This expectation to ‘endure silently’ helps explain persistent under-reporting and avoidance of healthcare, even among women experiencing severe physical or psychological symptoms [43,44].

Provider-side constraints also shaped survivors’ experiences. Several studies highlighted insufficient cultural sensitivity, limited preparedness for DV screening, and consultations characterized by rushed or transactional interactions. Women in Palestine reported feeling judged or unheard during brief encounters [45], while participants in Pakistan described staff behavior changing in the presence of others, undermining trust [46]. In Bangladesh, women avoided disclosure due to concerns about confidentiality [47]. Provider perspectives in Malaysia and Thailand reinforced these issues, with healthcare workers noting challenges in screening for DV within institutional routines influenced by workload, social norms, and limited training. Collectively, these findings illustrate how institutional factors, including time pressure, privacy constraints, and prevailing attitudes, can unintentionally reinforce provider bias and inhibit disclosure [48,49].

For example, in rural Northeast India, women reported experiencing obstetric violence during hospital births, highlighting how institutional practices can perpetuate harm even in settings intended for safety [50,51]. Similarly, in Malaysia and Palestine, survivors described dismissive attitudes and lack of privacy during consultations, discouraging future help-seeking [52].

The expectations survivors expressed were closely tied to culturally shaped understandings of care, authority, and gendered roles. Across contexts including Palestine, Bangladesh, Pakistan, Iran, and Malaysia, women consistently desired empathetic listening, respectful communication, and culturally competent support [41,53,54]. However, these expectations were often unmet due to systemic limitations and entrenched provider biases. Thematic synthesis further indicates that expectations for privacy, trauma-informed communication, and respect for autonomy were consistently reported across studies, reflecting broader regional patterns rather than isolated contexts [55,56].

An important insight from this review is that healthcare providers operate within the same cultural and social environments as survivors and are therefore influenced by similar norms surrounding domestic violence [57]. Studies indicate that providers in Asian contexts often face tension between professional responsibilities and cultural beliefs that frame violence as a private family matter, discouraging intervention [58]. This cultural embeddedness may limit providers’ willingness or capacity to screen for violence, respond empathetically, or initiate referrals, even when formal guidelines exist. In high-prevalence settings, some providers, particularly women, may also have personal or vicarious experiences of domestic violence, which may further influence clinical responses [59].

Without appropriate institutional support, supervision, and training, providers may experience emotional distress, avoidance, or moral conflict when encountering survivors, further constraining care delivery. The findings of this review therefore suggest that strengthening responses to domestic violence requires not only survivor-centered interventions, but also systematic support for providers, including trauma-informed training, clear clinical pathways, psychosocial support mechanisms, and workplace protections.

In parallel, broader public education and community-level interventions are needed to challenge norms that promote victim-blaming and silence, thereby reducing the cultural burden placed on both survivors and providers. Approaches that combine provider training, institutional accountability, and population-level awareness campaigns are more likely to create enabling environments where survivors can safely disclose abuse and providers can respond without risking social or professional repercussions [60].

Cultural theory helps illuminate this persistent mismatch between survivors’ needs and healthcare system responsiveness. Several included studies showed how biomedical routines prioritized visible injuries, while emotional, relational, and trauma-related harms received limited attention [33,38,61]. Survivors in Japan described long-term psychological consequences, somatic symptoms, and fragmented care pathways [25], while women in Sri Lanka and Pakistan similarly reported that emotional trauma was overlooked in clinical encounters [62]. These findings underscore the need to integrate trauma-informed principles validation, safety, and emotional support into routine care [63].

In collectivist contexts, decisions about seeking healthcare were often mediated by family power structures. Women in Bangladesh, India, and Iraq described how husbands, mothers-in-law, or male relatives discouraged healthcare use to avoid social embarrassment or preserve family honor [64]. Although some survivors received support from female relatives or community health workers, community-level stigma and patriarchal norms often intensified isolation and reduced autonomy [21,33]. These findings highlight the interplay between collectivist values, gender norms, and health-seeking behavior [33,65].

A consistent concern across studies was the limited readiness of healthcare systems to identify and respond to DV within culturally embedded environments. While some settings have introduced screening or referral mechanisms, survivors continued to encounter dismissive attitudes, inadequate referrals, and structural gaps, leading to missed opportunities for intervention, particularly in Palestine, Sri Lanka, Pakistan, and Malaysia [28,66,67]. These patterns suggest the need for culturally grounded, survivor-centered protocols that counter stigma, protect confidentiality, and support safe disclosure [68].

Survivors expected healthcare systems to provide safe, validating, and culturally respectful environments. In Thailand, for example, providers emphasized the value of IPV screening but identified cultural norms and limited training as barriers to creating safe spaces for disclosure [68]. These findings reinforce the importance of culturally sensitive, trauma-informed protocols that integrate privacy, empathy, and safety into routine care [69].

Across multiple settings, mental health needs emerged as critical and under-addressed. Women described symptoms of depression, anxiety, and trauma but rarely received appropriate psychological support, reflecting gaps in mental health integration. This pattern was evident in Japan, Sri Lanka, Pakistan, and Bangladesh, where survivors reported emotional distress that was not recognized or prioritized during clinical encounters [24]. Cultural stigma and limited availability of culturally appropriate mental health services further contributed to unmet needs [25,70].

Cultural theory helps explain these gaps by highlighting how norms related to emotional restraint, gender roles, and shame shape women’s help-seeking behavior [71]. In Japan, survivors described expectations to remain emotionally composed and avoid burdening others, limiting help-seeking [32]. Similarly, in Bangladesh and Pakistan, mental health services were often inaccessible or culturally discouraged due to stigma and lack of trained professionals [29,72]. As a result, many survivors turned to spiritual or informal sources of support, which did not fully address trauma [72].

Women also expressed a preference for integrated and holistic care models addressing physical, psychological, social, and legal impacts of domestic violence. Across contexts including Palestine, India, Pakistan, Sri Lanka, and Malaysia, survivors expected coordination between healthcare, social services, legal aid, shelters, and community networks [73]. However, systems were often described as fragmented, lacking clear referral pathways and intersectoral coordination, leaving survivors to navigate complex and disconnected services independently [73].

Applying cultural theory underscores the importance of contextualized care that recognizes the interwoven nature of health, social status, and cultural identity. Survivors’ expectations for holistic support reflect lived realities in which domestic violence affects multiple dimensions of life, from physical safety to economic stability and social belonging. In India, women emphasized the need for integrated access to legal services, counselling, shelter, and medical care [74].

Despite systemic challenges, several studies documented empowering and trust-building healthcare encounters. Survivors in Palestine, Bangladesh, Malaysia, and Korea described interactions marked by respectful communication, emotional support, and shared decision-making, which strengthened trust and encouraged future help-seeking [74,75]. These findings demonstrate that culturally competent, survivor-centered care can enhance women’s safety and recovery, even within constrained systems.

An important implication is the persistent lack of help-seeking among women experiencing domestic violence, despite high reported prevalence across Asian settings. Survivors frequently avoided or delayed seeking healthcare due to fear of stigma, retaliation, family repercussions, or previous negative experiences. As a result, many remain invisible to health systems, even when experiencing significant harm.

This pattern of non-disclosure suggests that existing estimates of domestic violence are likely underestimated, particularly in contexts where violence is normalized, reporting is discouraged, and confidentiality cannot be assured. Healthcare-based data capture only a portion of survivors’ experiences, typically those who reach crisis points or encounter supportive entry pathways. Strengthening trust, confidentiality, and culturally responsive care is therefore essential to improve outcomes and generate more accurate understanding of the true scale of domestic violence.

The evidence across included studies highlights key priorities for policy and practice. First, cultural competence and trauma-informed training should be integrated into healthcare curricula to enable providers to respond without judgement and with sensitivity to local norms [76]. Second, routine screening for intimate partner violence and accessible counselling services should be standard components of care, supported by safeguards for confidentiality and survivor autonomy [77]. Third, community-based interventions involving religious leaders, women’s groups, and cultural gatekeepers may help shift harmful norms, as sustainable change often emerges from within communities rather than external pressure [77]. Finally, expanding culturally adapted mental health services, strengthening intersectoral coordination, and reducing stigma are essential to improve outcomes and ensure comprehensive, affirming care for survivors [66,78].

## Limitations and future research

This review has several methodological limitations. First, full database-specific search strings were not archived, which limits the reproducibility and transparency of the search strategy. Second, although Google Scholar was used to broaden coverage, it may not comprehensively retrieve grey literature, particularly non-commercial sources such as government reports and policy documents. Third, screening and data extraction were not conducted independently by multiple reviewers, which may introduce selection bias. Additionally, the heterogeneity of included studies and the lack of longitudinal data limit the generalizability of the findings.

## Conclusion

This review highlights the strong influence of cultural norms and values on the healthcare experiences and expectations of women survivors of domestic violence in Asian contexts. Guided by cultural theory, the findings show how deeply rooted beliefs about gender roles, family honor, and social silence shape survivors’ interactions with healthcare systems. Women’s expectations for empathetic, confidential, and culturally sensitive care often remain unmet due to systemic gaps, provider biases, and institutional cultures that do not fully recognize the complexity of domestic violence.

To improve healthcare responsiveness, it is essential to integrate cultural competence into provider training, strengthen survivor-centred care models, and promote intersectoral collaboration. Addressing domestic violence in healthcare settings requires not only clinical interventions but also a clear understanding of the cultural contexts in which survivors live. By applying a cultural perspective, this review offers a useful framework for interpreting survivors’ needs and guiding policy and practice towards more inclusive and effective healthcare systems.

## Supplementary Material

PRISMA Checklist Resub.docx

## Data Availability

The data that support the findings of this study are available from the corresponding author upon reasonable request.

## Preprint

A preprint version of this manuscript has been posted on SSRN. The preprint has been cited in the manuscript [79].
